# Identification of Prophages in Bacterial Genomes by Dinucleotide Relative Abundance Difference

**DOI:** 10.1371/journal.pone.0001193

**Published:** 2007-11-21

**Authors:** K. V. Srividhya, V. Alaguraj, G. Poornima, Dinesh Kumar, G. P. Singh, L. Raghavenderan, A. V. S. K. Mohan Katta, Preeti Mehta, S. Krishnaswamy

**Affiliations:** Centre of Excellence in Bioinformatics, School of Biotechnology, Madurai Kamaraj University, Madurai, Tamilnadu, India; Weizmann Institute of Science, Israel

## Abstract

**Background:**

Prophages are integrated viral forms in bacterial genomes that have been found to contribute to interstrain genetic variability. Many virulence-associated genes are reported to be prophage encoded. Present computational methods to detect prophages are either by identifying possible essential proteins such as integrases or by an extension of this technique, which involves identifying a region containing proteins similar to those occurring in prophages. These methods suffer due to the problem of low sequence similarity at the protein level, which suggests that a nucleotide based approach could be useful.

**Methodology:**

Earlier dinucleotide relative abundance (DRA) have been used to identify regions, which deviate from the neighborhood areas, in genomes. We have used the difference in the dinucleotide relative abundance (DRAD) between the bacterial and prophage DNA to aid location of DNA stretches that could be of prophage origin in bacterial genomes. Prophage sequences which deviate from bacterial regions in their dinucleotide frequencies are detected by scanning bacterial genome sequences. The method was validated using a subset of genomes with prophage data from literature reports. A web interface for prophage scan based on this method is available at http://bicmku.in:8082/prophagedb/dra.html. Two hundred bacterial genomes which do not have annotated prophages have been scanned for prophage regions using this method.

**Conclusions:**

The relative dinucleotide distribution difference helps detect prophage regions in genome sequences. The usefulness of this method is seen in the identification of 461 highly probable loci pertaining to prophages which have not been annotated so earlier. This work emphasizes the need to extend the efforts to detect and annotate prophage elements in genome sequences.

## Introduction

Bacterial genomes evolve through a variety of process including horizontal gene transfer to survive under selective pressures exerted by the environment [1]. Internal modifications of genome by intergenomic homologous recombination and horizontal gene transfer (HGT) (intragenic recombination) have been prime reasons for bacterial genome diversity [2]. Mobile elements are responsible for the transfer of new functions to a bacterial cell and are recognized as important agents in bacterial evolution [3].

Bacteriophages (phage) are intracellular parasites that infect bacteria. Lytic phages upon infecting a cell, reproduce, lyse the cell and release progeny phages. However lysogenic or temperate phages multiply via the lytic cycle or enter a quiescent state in the cell. Prophages comprise of such DNA from phages in the integrated state. Fully functional prophages are capable of excision from the bacterial chromosome, either spontaneously or in response to specific signals particularly arising from damage to the host DNA. These lyse the host cells at some subsequent generation upon induction [4]. Prophages can also be defective (in a state of mutational decay and not induced to lytic growth) or be satellites (not carrying their own structural protein genes but capable of encapsidation by capsid proteins of other virions) [5].

Prophages can affect the fitness of the bacteria to survive. These, as elaborated by Brussow *et al*., 2004 [6] include (i) lysogenic conversion (ii) genome rearrangements, (iii) gene disruption, (iv) protection from lytic infection, (v) lysis of competing strains and (vi) introduction of new fitness factors (lysogenic conversion, transduction). Prophage–bacterial interaction has also been looked at from an ecological perspective by Chibani-Chennoufi *et al*., 2004 [7]. Such interaction becomes an essential survival strategy for both the prophage and the bacteria.

Prophages can constitute as much as 10–20% of a bacterium's genome and contribute to interstrain variability. The most extreme case is currently represented by the food pathogen *Escherichia coli* O157:H7 strain Sakai contains 18 prophage elements which amount to 16% of its total genome content [8], [9]. Many of these prophages are cryptic and in a state of mutational decay. Around 230 prophages are reported in 51 genomes [5]. Bacteriophages and prophages are major contributors of diversification in microbes [10]. The impact of prophages on bacterial chromosomes has been reviewed extensively [11] and it is seen that prophages are key agents for lateral gene transfer [12].

Prophages harbor virulence factors and pathogenicity islands, thereby playing an important role in the emergence of pathogens [13], [14]. This was recognized for diphtheria toxins and botulinum toxins, which are phage encoded. Virulence factor pertaining to prophage loci include toxins, pili (fimbriae), adhesins and secretion systems [6]. The CTXphi prophage of *Vibrio cholerae* encodes pathogenicity islands which it transfers into *Vibrio mimicus* . It has been pointed out that gain of virulence is not the only mechanism by which pathogenicity develops [15], [16]. In the prophage database (http://bicmku.in:8082) around 15 prophages are seen to encode virulence factors including toxin and adhesins, which contribute to pathogenicity in microbes [17].

### Prokaryotic genomes and associated fitness islands

Genomic islands increase the fitness of the bacterium. Such fitness islands are classified into several subtypes, such as ecological islands, saprophytic islands etc., based on their niche. These islands contribute to the host survival in the given environment. In many cases the fitness factor temporarily or permanently resides in the host either providing some benefits (‘Symbiosis islands’) or cause damage (pathogenicity islands (PAIs)) by interacting with living hosts. This flexible gene pool of bacteria is composed of prophages and other mobile elements or regions contrary to the core gene pool which comprises of the chromosomal segments pertaining to bacterial metabolic functions [18]. Pathogenicity islands are being explored quite frequently to understand disease development and evolution of bacterial pathogenesis [19]. The role of pathogenicity islands in the microbial evolution has been subject to extensive review [20], [21]. Yoon *et al* 2005 [22] have looked at 148 prokaryotic sequences and identified 77 candidate PAI's by applying a homology based method combined with abnormalities detected in genomic composition. Interestingly the same aspect could be looked at for understanding the evolution of eukaryotes by analyzing regions which deviate from the template DNA signature [18].

As reported by Brussow *et al*., 2004 [6], prophages harbor morons (more DNA), which provide extra fitness to the organism and are retained, imparting the bacterial host with some unique phenotype. Virulence factors have also been associated with prophages [15]. A database of bacterial virulence factors (VFs) associated with various medically significant bacterial pathogens is available. VFDB summarizes the conventional VFs (toxins, enzymes, cell-surface structures, such as capsular polysaccharides, lipopolysaccharides and outer membrane proteins, secretion machineries, siderophores, catalases, regulators) which directly or indirectly regulate pathogenesis in 16 important bacterial pathogens [23]. The mechanism of bacterial pathogenicity mediated by above VFs has been extensively studied by Wilson *et al* [17].

### Detection of genome heterogeneity

Heterogeneity in genomes is represented in many ways. Some of these include local and global variations in GC content, direct and inverted repeats, oligonucleotide relative abundance, genome mosaicism due to HGT, transposition and recombination events. Methods have been developed to identify potential foreign gene acquired by the bacterial genomes through horizontal gene transfer. A direct experimental method is subtractive hybridization. Comprehensive assessment of the extent of lateral gene transfer can be made easily by genomic subtraction, a procedure to enrich sequences that are present in one genome but not in another by using biotinylated subtractor DNA to fish out the target DNA by hybrid formation. Later after several cycles of hybridization with newly added subtractor DNA removes target DNA with sequences present in both target and subtracter strains. The remaining unbound target DNA is enriched in sequences absent in the subtracter DNA. This has been done for detecting lateral gene transfer, for example, in four strains of ***Salmonella enterica*** [24]. Indirect approaches include assessment of GC content, codon usage pattern and aminoacid usage [25], and dinucleotide relative abundance [26]. For example, HGT-DB is a repository of all the prokaryotic HGTs detected based on their deviation in G+C content, codon and amino-acid usage from prokaryotic complete genomes [27]. Genome heterogeneity in terms of short oligonucleotide compositional extremes and dinucleotide relative abundance distances between different parts of genomes have been examined by Karlin *et al*., 1994 [28]. This method focuses on small DNA sequences as an alternative to whole genome comparison methods and provides a meaningful measure of similarities. It has been observed that the dinucleotide relative abundance signature could discriminate local structure specificity more than sequence specificity. Dinucleotide relative abundance values are regarded as a stable property of DNA of an organism [25]. The method has been applied to phage genomes to understand similarities and dissimilarities associated with them. Compositional biases prevalent in bacterial genomes have also been examined by oligonucleotide distribution [29]. The significance of dinucleotide signatures in genome heterogeneity has been extensively reviewed by Karlin *et al* 1997 [30] in three facets namely, extremes of dinucleotide abundance, difference in genomic signatures in prokaryotes and evolution of genomes with respect to genomic signatures. Dinucleotide TA is seen to be under represented in eukaryotic genomes and not in viral and mitochondrial genomes. Contrarily, viral genomes are seen to be CG dinucleotide suppressed [25]. The transposable elements of *A thailana, C elegans D melanogoaster, H sapiens, S cerevisiae* display a similar pattern of relative abundance of dinucleotides in comparison with their respective host genomes [31]. This principle was extended over to prophage loci detection in microbial genomes.

### Prophage Identification methods in prokaryotic genomes

Recognizing prophages in bacterial genome sequences is not a straight-forward task as prophage sequences are mosaic and encode many orphan and hypothetical proteins, hence unambiguous identification is difficult. Extensive work has been done for detecting ‘corner stone genes’ for the purpose of identifying prophages in bacterial genomes. Integrases are usually sufficiently conserved to be recognizable. Although most temperate phages have an integrase gene, it is not a necessary and sufficient condition to prove the existence of a prophage [5]. Prophages do harbor some phage virion assembly proteins such as Terminase, Portal protein, Head maturation protease, Coat protein, Tail tape measure protein.

A comprehensive bioinformatic analysis was earlier carried out for the e14 cryptic prophage sequence [32]. This showed that the e14 is modular and shares a large part of its sequence with *Shigella flexneri* phage SfV [32]. Based on this similarity, the regulatory region including the repressor and Cro proteins and their promoter binding sites were identified. A protein based comparative approach using the COG database as a starting point was carried out to detect new lambdoid prophage like elements in a set of completely sequenced genomes [32]. This protein similarity approach (PSA) was extended by the use of BLAST similarity searches rather than limiting to the COG database [33], [34]. The PSA method was tested with bacterial genomes having known reports of prophages and then extended to newly sequenced bacteria. A total of 87 prophage loci could be identified from 61 bacteria [33], [34]. Bose and Barber 2006 [35] have implemented prophage loci prediction tool for prokaryotic genome sequences based on BLASTX sequence comparison against phage proteomes. Subsequently, a heuristic automated program proposed by Fouts 2006 [36] for prophage detection enables multiple curation of identified prophage locus by comparison with HMMs of phage proteins and further facilitates sub classification of the identified locus.

Dinucleotide Relative abundance (DRA) approach takes into account the local heterogeneity within the given bacterial genomes. DRA values are reported to remain relatively uniform within a genome and its closely related organisms. On this basis, the collection of sixteen DRA values has been referred to as a genomic signature. Thus local heterogeneity in DRA values has been used to detect alien regions in bacterial genomes [25]. This method has also been applied to phage genomes to understand similarities and dissimilarities associated with them [29]. We have modified this approach to detect prophages in bacterial genomes. Putative prophage regions could be identified by finding local regions of bacterial genomes that show significant deviation in dinucleotide abundance relative to the background. However, these regions should also show similar dinucleotide abundance relative to that of a reference set of non redundant prophage sequences relevant for those bacteria. Hence taking a dinucleotide relative abundance difference (DRAD), with reference to the two cases described, improves the ability to detect the deviant regions. Since not all the dinucleotides show variation, an appropriate selection helps to further increase the discrimination of the prophage regions.

## Results and Discussion

A program to detect prophage regions (both functional and prophage remnants or highly defective prophages) was developed based on comparison of DRAD analysis. From a total of 52 genomes, 325 probable prophage loci could be identified. Of these 95 prophage loci were earlier reported in literature (Table 1). The rest 230 were newly identified loci among which 159 were highly probable loci. Details are available at http://bicmku.in:8082/prophagedb/newprophages.html.

**Table 1:** Prophages identified using dinucleotide relative abundance difference method.

Bacterial genome	Known prophages		new prophages detected by DRAD	Comment/phenotype/Infection
	Reported in literature	Also found by DRAD		
Brucella suis 1330 *	1	0	5	Intracellular pathogen and potential bioterrorism agent,
Clostridium tetani E88 *	3	0	1	tetanus
*Deinococcus radiodurans R1 #*	2	1	2	radiation-resistant bacterium
Escherichia coli 0157:H7EDL933*	20	19	11	hamburger-borne and hemolytic uremic syndrome
Escherichia coli 0157:H7sakai*	24	23	6	diarrhea, haemorrhagic colitis, and haemolytic uremic syndrome.
Escherichia coli CFT073*	8	6	14	uropathogenic
*Escherichia coli K-12*	10	8	5	commensal
Haemophilus influenzae Rd KW20 *	3	0	6	cellulitis, osteomyelitis, epiglottitis,
*Lactococcus lactis IL1403*	6	1	2	dairy industry as starters for cheese making
Listeria innocua CLIP1162 *	6	0	3	listeriosis
Listeria monocytogenes EGD-e *	2	0	6	listeriosis
*Mesorhizobium loti MAFF303099 #*	3	0	6	nitrogen-fixation
Mycobacterium tuberculosis CDC1551*	2	0	1	Tuberculosis
Neisseria meningitidis MC58 *	2	0	5	meningitis and septicemia
Neisseria meningitidis Z2491 *	3	0	4	meningitis and septicemia
*Oceanobacullus iheyensis HTE831 #*	1	0	3	halotolerant and alkaliphilic
Pseudomonas aeruginosa PAO1 *	2	1	4	opportunistic human infections
*Pseudomonas putida KT2440*	4	1	7	degrade organic solvents
Ralstonia solanacearum GMI1000 *	8	1	2	plant pathogen
Salmonella enterica CT18 Serovar Typhi*	11	7	10	typhoid fever
Salmonella enterica Serovar Typhi ty2*	7	7	8	typhoid fever
*Salmonella entericaLT2 Serovar Typhimurium*	7	4	5	typhoid fever
*Shewanella oneidensis MR–1*	3	0	7	metal ion-reducing bacterium
Shigella flexneri 2a 301 *	11	8	9	bacillary dysentery or shigellosis
Staphylococcus aureus Mu50 *	3	0	1	toxic-shock syndrome and staphylococcal scarlet fever,
Staphylococcus aureus N315 *	1	1	1	toxic-shock syndrome and staphylococcal scarlet fever,
Streptococcus agalactiae 2603 V/R *	2	0	2	invasive neonatal disease
Streptococcus pyogenes M1 SF370 *	4	0	1	rheumatic fever or acute glomerulonephritis
Streptococcus pyogenes M18 MGAS8232 *	5	2	1	Acute rheumatic fever (ARF), a sequelae of group A Streptococcus (GAS) infection
Streptococcus pyogenes M3 MGAS315 *	6	1	1	a sequelae of group A Streptococcus (GAS) infection
Vibrio cholerae N16961*	2	0	3	cholera pathogen
Xanthomonas axonopodis 903 *	2	1	5	citrus cankers and black rot
Xanthomonas campestris ATCC33913 *	3	0	7	black rot
Xylella fastidiosa 9a5c *	9	0	3	citrus variegated chlorosis
Xylella fastidiosa Temecula *	8	0	4	citrus variegated chlorosis

Pathogenic organisms are indicated in * and organism surviving on varied ecological niche/having industrial significance are indicated in #. DRAD refers to the method reported here.

The sensitivity and specificity of the method was found to average around 82% and 83% respectively (Table 2) but however varied amongst different genomes. Our analysis suggests that the variation is not related to the GC content. The variation is possibly related to the non redundant nature of the prophage set used for the detection.

**Table 2:** Sensitivity and Specificity across genomes.

Bacterial genome	DRAD	literature (lit)	overlap DRAD+lit	Evidenced from annotation	TP	FN	FP	Sn	Sp
*Deinococcus radiodurans R1*	3	2	1	2	3	1	0	0.75	1.00
*Escherichia coli 0157:H7EDL933*	38	20	19	11	30	1	8	0.97	0.79
*Escherichia coli 0157:H7sakai*	32	24	23	6	29	1	3	0.97	0.91
*Escherichia coli CFT073*	24	8	6	14	20	2	4	0.91	0.83
*Escherichia coli K-12*	17	10	8	5	13	2	4	0.87	0.76
*Lactococcus lactis IL1403*	4	6	2	2	4	4	0	0.50	1.00
*Pseudomonas aeruginosa PAO1*	5	2	1	4	5	1	0	0.83	1.00
*Pseudomonas putida*	8	4	1	7	8	3	0	0.73	1.00
*Ralstonia solanacearum*	3	8	1	2	3	7	0	0.30	1.00
*Salmonella enterica CT18 Serovar Typhi*	23	11	7	10	17	4	6	0.81	0.74
*Salmonella enterica Serovar Typhi ty2*	19	7	7	8	15	0	4	1.00	0.79
*Salmonella entericaLT2*	17	7	4	5	9	3	8	0.75	0.53
*Staphylococcus aureus N315*	2	1	1	1	2	0	0	1.00	1.00
*Streptococcus pyogenes M18 MGAS8232*	3	5	2	1	3	3	0	0.50	1.00
*Streptococcus pyogenesM3 MGAS315*	3	6	1	1	2	5	1	0.29	0.67
*Streptococcus agalactiae 2603 V/R*	3	2	2	1	3	0	0	1	1
*Shigella flexneri 2a 301*	17	11	8	9	17	3	0	0.85	1.00
*Xanthomonas axonopodis 903*	6	2	1	5	6	1	0	0.86	1.00

Comparision of prophage locus detected by DRAD against literature reported and evidence from annotation. DRAD refers to the method reported here.

TP–Probable True postivies, FN–false negatives , FP-False positives , Sn–Probable Senstivity, Sp-Probable Specificity

**Figure 1:**
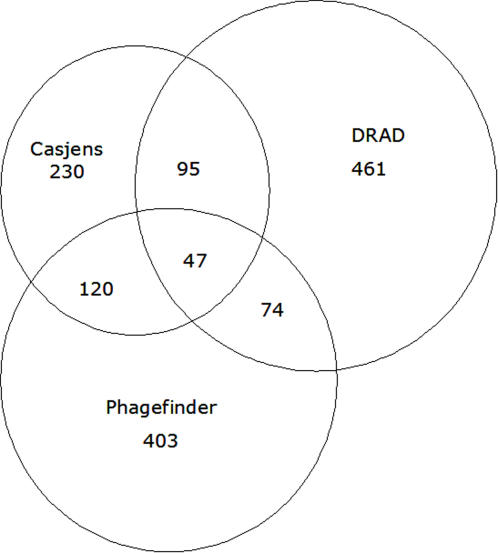
Comparative analysis of number of prophages identified by the approach reported here (DRAD), literature reports and another prophage detection method (phage_finder tool).

**Figure 2:**
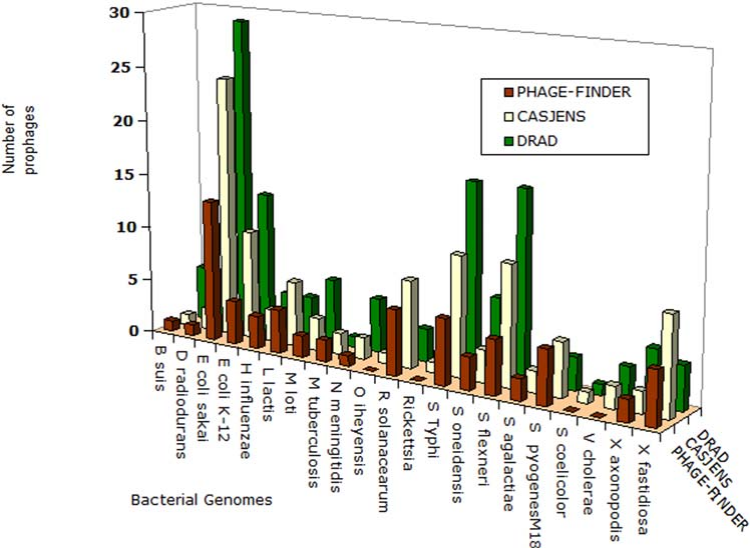
Variation of prophage number with bacterial genomes. – Indicated in green are prophages identified by the method reported here (DRAD), yellow and red represents prophage loci reported in literature [5] , identified by phage_finder program [35] respectively.

A comparison between the prophages identified by our method, those reported by Casjens [5] and a method phage_finder [35] shows a common overlap of 47 prophages (Figure 1 and Figure 2). The details on the prophage loci reported by different methods are given at http://bicmku.in:8082/prophagedb/prophage_different_methods.htm. The detection of prophages varies between different genomes suggesting that it would be necessary to use more than one method depending on the genome in order to locate all possible prophages. This probably arises from the mosaic nature of prophages.

### Bacterial genomes with no earlier report of prophages

**Figure 3:**
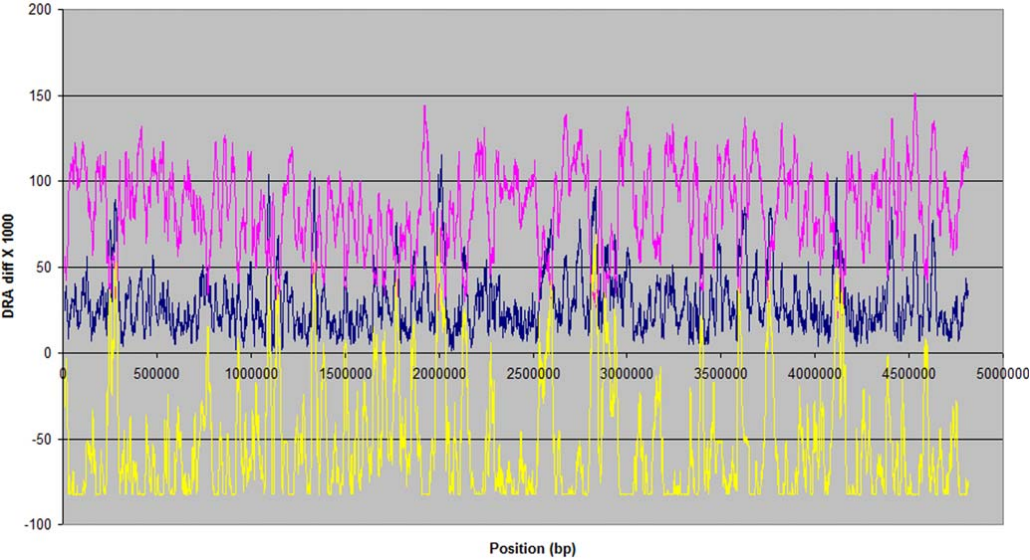
Dinucleotide difference distribution for *Shigella sonnei:* pink-*Shigella sonnei* genome Vs *Shigella sonnei* genome, blue-*Shigella sonnei* genome Vs prophage dataset , yellow- their dinucleotide relative abundance difference (DRAD) value.

The DRAD method was used to examine genome sequences with no reports of prophages. A total of 200 genome sequences were analyzed for prophage elements using this DRAD approach. Out of the 453 loci identified from 84 bacterial genomes, 207 (from 64 genomes) were seen to be highly probable prophage loci, based on the annotation in the protein table files of the corresponding bacterial genomes. The genome of *Shigella sonnei* had high incidence of thirteen prophages (Figure 3) http://bicmku.in:8082/prophagedb/patho_prophages.html.

### Prophages in bacterial genomes with varied ecological niche

The acquisition of ecological islands by the bacterial host occurs through horizontal gene transfer [18]. A total of 96 prophage loci could be identified form 35 bacterial genomes (Table 3) which grow in extreme ecological niches or are being exploited for industrial production. The detailed loci of the prophages are available at http://bicmku.in:8082/prophagedb/eco_prophages.html.

**Table 3:** Prophages associated with bacterial genomes surviving on varied ecological niches/with industrial significance.

Bacteria	Comment on phenotype	Prophage hits	Proteins encoded by prophage
*Bacillus clausii KSM-K16*	Endosymbiont	3	Phage proteins and morons
*Bacteroides thetaiotaomicron VPI-5482*	Endosymbiont	1	Transposase and type II systems
*Bradyrhizobium japonicum USDA 110*	Nitrogen fixing bacterium	3	Transposase , integrase
*Chlorobium tepidum TLS*	Thermophilic green sulfer bacteria	1	Secretion systems
*Colwellia psychrerythraea 34H*	Psychrophilic	3	Glucosyl transferase , transposase
*Corynebacterium efficiens YS-314*	Industrial organism	1	Capsule proteins
*Dehalococcoides ethenogenes 195*	Dechlorinate ground water	3	Virulence , HNH , recombinase, integrase and transposase
*Desulfovibrio vulgaris*	Bioremediation of toxic metal ions	5	Phage proteins, restriction systems and transposase
*Frankia sp. CcI3*	Nitrogen-fixing bacterium	1	Excisionase
*Geobacillus kaustophilus HTA426*	Thermophilic	9	Phage proteins, Transposase , recombinase and restriction systems
*Geobacter sulfurreducens PCA*	Environmental restoration	1	Transposase and glucosyl transferase
*Hahella chejuensis KCTC 2396*	Algicidal pigment	8	Phage, flagellar-pilus proteins, glucosyl transferase
*Lactobacillus sakei subsp. sakei 23K*	Biopreservation and food safety	2	Transposase and glucosyl transferase
*Rhodopseudomonas palustris HaA2*	Phototrophic bacterium	1	Phage proteins
*Rhodospirillum rubrum ATCC 11170*	Photosynthetic bacterium	1	Resolvase, intergrase and capsid proteins
*Salinibacter ruber DSM 13855*	Hyperhalophilic Archaea	1	Transposase, integrase, morons
*Zymomonas mobilis subsp. mobilis ZM4*	Industrial organism	2	Restriction modification systems and transposase

### Pathogenicity islands and prophages

The role of bacteriophages contributing to pathogenicity has been reviewed by Tinsley *et al*., 2006 [3]. Prophage loci are seen to encode pathogenicity islands. This study showed that in the 29 pathogenic bacterial genomes screened (Table 4), 207 prophage loci were identified. Of these, 111 were seen to encode virulence or fitness factors. Details of the loci are available at http://bicmku.in:8082/prophagedb/patho_prophages.html. The observations suggest that acquisition of virulence genes through horizontally transferred prophages could be a common strategy of microbes undergoing transformation from a commensal to a pathogen. With the availability of bacterial genomes sequences, it is evident that inter-species transmission of genetic information is pervasive in microbes and that parallely acquisition of foreign genes is counter balanced by loss of native genes, in order to maintain genome size within limits.

**Table 4:** Prophage loci, in pathogenic bacteria, identified by the method reported here (DRAD approach) indicated as * are PAIs reported by Yoon *et al* 2005 [22].

Bacterial genome	Prophage loci	Infection	Gene products/Fitness factor
*Bacillus anthracis str. Ames*	2	Anthrax bacterium	MORONS-glucosyl transferase
*Bacillus cereus ATCC 10987*	1	Food poisoning	MORONS-glucosyl transferase
*Bacillus thuringiensis serovar konkukian str.*	3	Insceticidal	Flagellar and sporulation proteins
*Bacteroides fragilis NCTC 9343*	1	Severe GI infections	Transposase
Bordetella pertussis Tohama I*	3	Whooping cough	Transposase , amidase and type II systems
*Brucella abortus biovar 1 str. 9-941*	3	Brucellosis and undulant fever	Transposase
*Burkholderia pseudomallei 1710b*	3	Melioidosis	Restriction systems , transposase and phage proteins
*Burkholderia pseudomallei K96243*	1	Melioidosis	Restriction systems , transposase and phage proteins
Chromobacterium violaceum ATCC 12472*	1	Pathogenic and industrial	Glucosyl transferase and lysis protein
*Corynebacterium diphtheriae NCTC 13129*	1	Diphtheriae	Phage and HNH proteins
*Coxiella burnetii RSA 493*	1	Q fever	Pilus proteins
*Erwinia carotovora subsp. atroseptica*	7	Soft rot and blackleg potato diseases	Phage, flagellar-pilus proteins , integrase
*Haemophilus ducreyi 35000HP*	2	Chancroid	Phage and repressor proteins
Helicobacter pylori J99*	1	Peptic ulcer	CAG island protein(pathogenicity)
*Leptospira interrogans serovar copenhageni str. Fiocruz L1-130*	2	Leptospirosis	Transposase and outer membrane proteins
*Leptospira interrogans serovar Lai*	2	Leptospirosis	Glucosyl transferase and fimbrial proteins
*Mycobacterium avium K10*	3	Mycotic Diseases	Lysis protein
*Mycobacterium bovis AF2122/97*	3	Tuberculosis	Antigenicity associated protein
Photorhabdus luminescens TT01*	9	insect-pathogenic bacterium	Virulence sensor protein, transposase and IS elements
*Pseudomonas syringae pv. phaseolicola*	5	brown spot halo light of tomato	Transposase, pilus protein and glucosyl transferase
*Salmonella enterica subsp. enterica serovar Choleraesuis str. SC-B67*	11	Salmonellosis, swine paratyphoid	Fimbrial and usher proteins(virulence), secretion systems, glucosyl transferase
*Salmonella enterica subsp. enterica serovar Paratyphi A str. ATCC 9150*	9	Relapsing fever	Pathogenicity island and secretion system , fimbrial, O antigen protein,integrase ,
*Shigella boydii Sb227*	11	Dysentery	Phage proteins, glucosyl transferase fimbrial proteins, drug resistance protein and IS elements
*Shigella dysenteriae Sd197*	5	Dysentery	Phage proteins,drug resistance protein,IS and sidephore related proteins
*Shigella sonnei Ss046*	13	Mucoid diarrhea	Phage proteins,lysis casette, integrase , glucosyl transferasedrug resistance protein,IS and sidephore related proteins
*Streptococcus pyogenes MGAS5005*	1	a sequelae of group A Streptococcus (GAS) infection	Mostly phage proteins
*Treponema denticola ATCC 35405*	1	Periodontal disease	Hydrolase
Vibrio parahaemolyticus RIMD 2210633*	2	Gastrointestinal disease	Pilus assembly protein and restriction proteins
*Yersinia pseudotuberculosis IP 32953*	4	Mesenteric adenitis	Phage and fimbrial proteins

The DRAD analysis carried out with *Bacillus anthracis* showed two prophage loci that encode morons (glucosyl transferase). This supplements the report of four prophages being associated in *B anthracis* by Sozhamannan *et al*., 2006 [37] . *Erwinia carotovora* subsp. *atroseptica* is an important bacterial plant pathogen causing soft rot and blackleg in potato. As a member of the Enterobacteriaceae, it is related to *Escherichia* and *Shigella*, *Salmonella* and *Yersinia* [38]. In this study, *Erwinia* was found to harbor a total 7 prophages encoding Type IV pilus protein and flagellar proteins. Similarly, in the pathogenic *H pylori* genome, the DRAD analysis identified prophage loci that encode Cag island proteins which pertain to pathogenicity [39]. The same Cag island has been reported by Yoon *et al*., 2005 [22] as potential PAI. Moreover, in *Chromobacterium violaceum ATCC 12472 , Bordetella pertussis Tohama I, Helicobacter pylori J99, Photorhabdus luminescens TT01 Vibrio parahaemolyticus RIMD 2210633* (Table 4) the prophage loci identified by DRAD compare well with the PAIs reported by Yoon *et al*., 2005 [22].

In the case of *Mycobacterium avium the* prophage region detected by DRAD was found to encode MurA, which has been implicated in *M. tuberculosis* resistance to a range of broad-spectrum antimicrobial agents [40]. With *Mycobacterium bovis out* of three prophages that were detected one was found to harbor PE-PGRS genes, which are a family encoding numerous repetitive glycine-rich proteins of unknown function [41]. PE-PGRS proteins are reported to be associated with mycobacterial species (*M*. *tuberculosis, M. bovis BCG, M. smegmatis, M. marinum and M. gordonae*) and 11 clinical isolates of M. tuberculosis [42]. This again highlights the possible contribution of prophages to the virulence of the associated bacterial species.

*Salmonella enterica subsp. enterica serovar Choleraesuis* is a highly invasive serovar among non-typhoidal Salmonella that usually causes sepsis or extra-intestinal focal infections in humans [43]. The DRAD analysis of the bacterial genome showed a high incidence of prophages. The loci identified encode Gifsy-2 and Gifsy-1 prophage like proteins. Most of loci encode a few to many fimbrial proteins, surface presentation antigens and secretion system apparatus which are key genes involved in virulence. In the case of *Salmonella enterica Paratyphi*, a human-restricted serovars of Salmonella enterica causing typhoid [44], nine prophage loci could be identified and these predominantly encode pathogenicity islands apart form secretion systems.

Maurelli *et al* 1998 [45] have reported the role of genomic deletion (of LCD- lysine decarboxylase) contributing to the pathogenicity of *Shigella* spp. Among *Shigella* species, *S sonnei* involved in mucoid diarrhea, 13 highly probable prophage loci could be detected. With all the three species of *Shigella* (S. s*onnei*, S.*boydii* and S.*dysenteriae*) almost all the loci are associated with insertion sequence elements, from a minimum of 3 to 10. A few of the possible prophage loci are seen to harbor virulence factors like siderophores. In *Vibrio parahaemolyticus*, the two prophage loci that have been detected (Table 4) encode pilus assembly protein and restriction proteins. Recently, horizontal gene transfer of CTXphi prophage encoded PAIs have been reported between *V mimicus* and *V cholerae* [46] indicating that the *Vibrios* share such virulence associated gene pools.

### Conclusion

Prophages, including defective ones, can contribute important biological properties to their bacterial hosts. In order to understand completely the nature of the bacterial behavior, one must be able to recognize the full complement of prophages in bacterial genomes. The extreme variability of prophage sequences, as seen by our comparisons, makes it quite possible that unrecognized prophages are still present in bacterial genome sequences (Casjens, 2003)[5] .We have presented a dinucleotide distribution difference method for identification of prophages from microbial genomes sequences. Prophage detection methods such as the one described here based on dinucleotide composition and those earlier reported based on similarity at the protein level tend to supplement each other. With increasing microbial genome sequences being available, consensus methods will probably emerge for identifying potential prophage loci in microbial genomes. These will help explain the prophage mediated evolution of microbes.

## Materials and Methods

The Dinucleotide Relative Abundance (DRA) [28] was modified for prophage detection.

For a given dinucleotide XY,
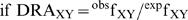
(1)where ^obs^f_XY_ is the observed frequency of the dinucleotide XY occurring in a chosen window and ^exp^f_XY_ is the expected frequency of the nucleotide XY occurring in the reference set.
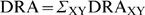
(2)DRA^bact^ is calculated using the observed dinucleotide frequencies for a window of the bacterial genome and the expected frequencies of the dinucleotide occurring over the entire bacterial genome. The DRA^bact^ values using a sliding window are calculated for the entire genome and plotted against the bacterial genome sequence position. DRA^prophage^ is calculated using the observed dinucleotide frequencies for a window of the bacterial genome and the expected frequencies of the dinucleotide occurring over the entire prophage reference set. The DRA^prophage^ values using a sliding window are calculated for the entire genome and plotted against the bacterial genome sequence position.
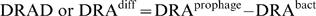
(3)The DRAD or DRA^diff^ is calculated for each window and plotted against the bacterial genome sequence position. Regions of high DRA^diff^ values are used to identify possible prophage-like regions. By trial and error, using known prophage regions, a window size of 25000 with a displacement of 1000 was standardized for the screening. Further the hit was annotated as a potential prophage locus and taken as a true positive if the annotation in protein table (ptt) file for the locus had phage associated genes. Those regions without any phage marker genes were considered as false positives. The annotations of peak locus (corresponding to each prophage) were retrieved from protein table file (ptt) of respective bacterial genomes. False negatives includes prophage set not detected by DRA but reported in literature.

The probable specificity (ratio of true positives to the sum of true positives and false positives) and probable sensitivity (ratio of true positives to the sum of true positives and false negatives) were calculated according to Makarov 2002 [47]. The qualifier probable has been added to the specificity and sensitivity measures since the assumption that the data used for validation is complete is not wholly appropriate, as there could be prophages that are yet to be detected. A server for the detection of prophages based on comparison of Dinucleotide Relative Abundance Difference (DRAD or DRA^diff^) values is available at http://bicmku.in:8082/prophagedb/dra.html.

### Data Source

Bacteria genomes were downloaded from NCBI ftp site (ftp://ftp.ncbi.nih.gov/genomes/Bacteria/). Prophage positions and sequences obtained from supplementary material of Casjens, (2003) [5] are available in the prophage database (http://bicmku.in:8082/prophagedb, Srividhya et al 2006) [34]. Location of prophages in bacterial genomes was determined by using protein table file (ptt) from NCBI.

### Construction of Non-redundant Prophage set (NRPS)

For detection of new prophages in bacterial genomes a set of non redundant prophages was constructed, which includes prophages (without repetition) from 50 bacterial genomes from the prophage database (http://bicmku.in:8082). This constitutes the NRPS (non-redundant prophage set) which was used for screening for prophages in any given bacterial genome. The list of prophages taken for NRPS generation is listed in http://bicmku.in:8082/prophagedb/nrlist.html.
